# Adaptive Servo-Ventilation for Central Sleep Apnea in an Anemic Patient with Cardiac Disease: A Case Report

**DOI:** 10.3390/reports8030140

**Published:** 2025-08-07

**Authors:** Bianca Domokos-Gergely, Gabriel-Flaviu Brișan, Doina Todea

**Affiliations:** 1 “Leon Daniello” Clinical Hospital of Pneumophysiology, 400371 Cluj-Napoca, Romania; 2 Department of Medical Sciences—Pulmonology, Faculty of Medicine, Iuliu Hațieganu University of Medicine and Pharmacy, 400012 Cluj-Napoca, Romania

**Keywords:** Cheyne–Stokes breathing (CSB), central sleep apnea (CSA), obstructive sleep apnea (OSA), continuous positive airway pressure (CPAP), adaptive servo-ventilation (ASV), anemia

## Abstract

**Background and Clinical Significance**: Obstructive sleep apnea (OSA) is a common comorbidity in patients with cardiac and metabolic disorders. The coexistence of central sleep apnea with Cheyne–Stokes breathing (CSA-CSB) in heart failure patients, especially those with preserved ejection fraction (HFpEF), represents a diagnostic and therapeutic challenge. Data on continuous positive airway pressure (CPAP) failure and successful adaptation to servo-ventilation (ASV) in the context of complex comorbidities remain limited. **Case Presentation**: We present the case of a 74-year-old male with a history of type 2 diabetes mellitus, paroxysmal atrial fibrillation, HFpEF, essential hypertension, and bladder carcinoma. He was referred for pre-operative OSA screening, reporting excessive daytime sleepiness, insomnia, and witnessed apneas. Initial respiratory polygraphy revealed severe sleep-disordered breathing with dominant CSA-CSB and moderate OSA. Laboratory investigations also revealed severe iron-deficiency anemia, which was managed with parenteral iron supplementation. The patient underwent CPAP titration, which led to modest improvement and residual high apnea–hypopnea index (AHI). After persistent symptoms and an inadequate CPAP response, an ASV device was initiated with significant clinical and respiratory improvement, demonstrating normalization of hypoxic burden and optimal adherence. **Conclusions**: CSA-CSB in HFpEF patients with anemia poses unique therapeutic difficulties. This case highlights the importance of individualized diagnostic and therapeutic strategies, including transitioning to ASV in CPAP-refractory cases, which can lead to improved adherence, reduced hypoxia, and better overall outcomes in high-risk patients.

## 1. Introduction and Clinical Significance

Central sleep apnea with Cheyne–Stokes breathing (CSA-CSB) poses a significant clinical challenge, particularly in patients with overlapping conditions such as obstructive sleep apnea (OSA), atrial fibrillation, heart failure, and neurological disorders. In heart failure with preserved ejection fraction (HFpEF), the prevalence of CSA-CSB ranges from 18% to 30%, increasing with the severity of diastolic dysfunction. OSA sometimes coexists with a CSA component, which is observed in approximately 12.2% of heart failure patients. The presence of CSA-CSB is associated with a worse hemodynamic profile and prognosis compared to patients with HFpEF and OSA alone. It remains unclear whether CSA-CSB is merely a marker of advanced cardiac dysfunction or if it independently contributes to cardiac remodeling through mechanisms such as heightened sympathetic activity [1,2,3].

The pathogenesis of CSA-CSB is primarily attributed to ventilatory instability, often conceptualized through the loop gain model; in addition to high loop gain, a narrow CO_2_ reserve also plays a role in ventilatory control instability. This results in the cyclical pattern of hyperpnea and apnea observed in CSA-CSB. Anemia may further exacerbate this instability by reducing oxygen-carrying capacity, potentially increasing chemosensitivity and contributing to the persistence of CSA-CSB [3,4].

Full polysomnography with esophageal pressure monitoring remains the gold standard for diagnosing CSA-CSB, though surrogate methods are often used in practice. Management begins with optimizing heart failure treatment—reducing congestion, sympathetic drive, afterload, and controlling arrhythmias. If CSA-CSB persists, positive airway pressure therapies are considered. While continuous positive airway pressure (CPAP) may be effective in some cases, others continue to exhibit periodic breathing. In such cases, adaptive servo-ventilation (ASV) may be indicated. Contributing factors like anemia can exacerbate respiratory instability, leading to poor sleep quality, CPAP intolerance, and reduced quality of life [2,3].

This case report aims to shed light on the real-world challenges of managing a patient with multiple coexisting health issues, including central sleep apnea with Cheyne–Stokes breathing (CSA-CSB). Through a detailed account of the diagnostic process and the challenges encountered during titration studies, we aim to show how clinical decisions often need to be tailored when standard treatments meet complex patient profiles and overlapping sleep-related breathing disorders.

## 2. Case Presentation

### 2.1. Initial Presentation, Diagnosis, and CPAP Titration

A 74-year-old male, former smoker (20 pack-years), was referred to our clinic for a pre-operative assessment of obstructive sleep apnea (OSA), following a routine STOP-BANG questionnaire screening, as part of the standard pre-anesthesia evaluation recommended by the Anesthesia and Intensive Care team.

The patient has a history of type II diabetes mellitus, paroxysmal atrial fibrillation, essential hypertension, and bladder carcinoma, for which transurethral resection of bladder tumor (TURBT) was planned. He also reported intermittent macroscopic hematuria.

The patient was under treatment with angiotensin-converting enzyme inhibitor (IECA), diuretics and beta-blockers, and oral anticoagulant, alongside antidiabetic therapy with metformin and sulfonylureas.

Sleep-related symptoms included excessive daytime sleepiness (Epworth Sleepiness Scale: 15/24), mixed insomnia (difficulty initiating and maintaining sleep), and nocturnal apneic episodes witnessed by his wife. He denied the use of alcohol, sedatives, or illicit drugs. The STOP-BANG score was elevated (7/8), indicating a high risk for obstructive sleep apnea (OSA).

Clinical examination revealed abdominal obesity, a BMI of 34 kg/m^2^, Mallampati class III, pallor of the conjunctivae and palms, blood pressure of 113/68 mmHg, and regular tachycardia at 114 bpm. Electrocardiography (ECG) showed sinus tachycardia without ischemic changes.

Respiratory and pulmonary assessment included normal diurnal arterial blood gases (pH 7.44, pCO_2_ 36 mmHg, pO_2_ 78 mmHg, HCO_3_^−^ 25.4 mmol/L). Pulmonary function testing revealed a mild restrictive ventilatory defect with a preserved FEV_1_/FVC ratio (>0.7), specifically, a FVC of 2.88L (78%).

Laboratory investigations showed moderate normocytic, hypochromic anemia (Hb 8.9 g/dL), with anisocytosis and poikilocytosis on peripheral smear and a low ferritin level, consistent with iron-deficiency anemia.

Cardiology consultation confirmed compensated heart failure with preserved ejection fraction of 55% (HFpEF), essential hypertension, and sinus tachycardia, excluded the presence of intracardiac thrombus, and advised discontinuation of oral anticoagulant therapy until the hematuria resolves. Continuation of loop diuretics and beta-blockers was advised, with initiation of an SGLT-2 inhibitor planned after resolution of the hematuria and stabilization of blood pressure, with close monitoring of vital signs.

Parenteral iron therapy with iron sucrose (100 mg daily for 6 days) was initiated due to the patient’s refusal of blood transfusion, with dosing adjusted according to hemoglobin level and weight, followed by oral iron supplementation after discharge. Additionally, intravenous Ringer’s solution was administered as supportive therapy. Urology consultation for macroscopic hematuria recommended TURBT and parenteral carbazochrome and etamsylate.

Due to a high suspicion of OSA and the presence of multiple comorbidities, a type II sleep study–polysomnography (PSG), unattended in a sleep lab, was initially indicated. However, as the patient declined head sensors, a type III sleep study–respiratory polygraphy (RP) was instead performed in the hospital using the SleepDocPorti 9 system. This recorded nasal airflow, heart rate, SpO_2_, body position, and thoracoabdominal movements. The data were manually scored by a sleep specialist according to AASM guidelines.

**Figure 1:**
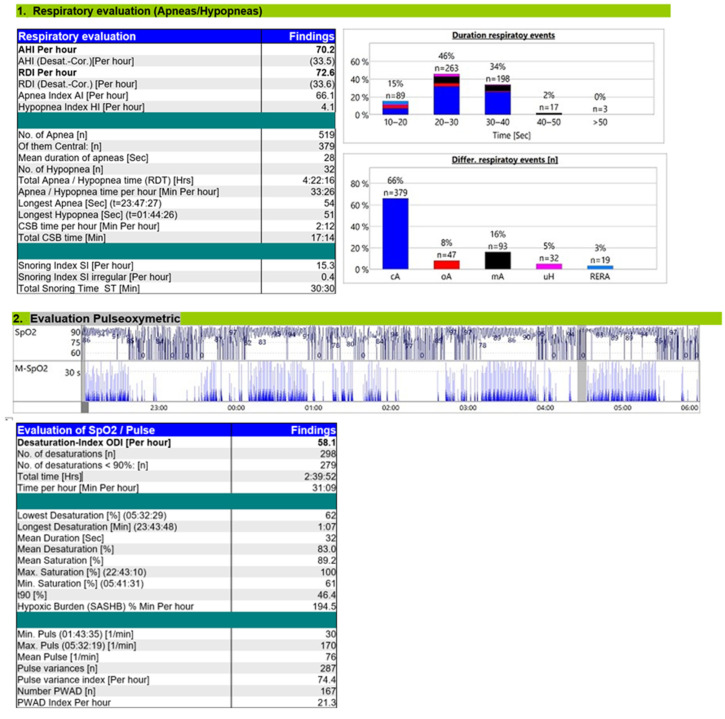
Diagnostic RP report.

**Figure 2:**
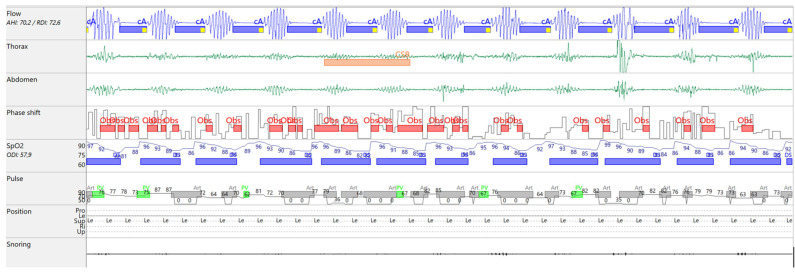
Flow tracing on MiniScreen Viewer, Cheyne–Stokes breathing pattern.

The diagnostic RP (Figure 1) revealed severe CSA with CSB (Figure 2) and a moderate OSA component. The overnight RP was of good technical quality, with a validated recording time of 7 h and 50 min, ensuring a reliable analysis. The sleep study confirmed the diagnosis of severe central sleep apnea (CSA), with an overall apnea–hypopnea index (AHI) of 70.2 events/h of recording. A total of 519 apnea events were recorded, of which central origin predominated (379 events; 73%), with a final central AHI of 48.3 events/h of recording. A characteristic crescendo–decrescendo respiratory pattern consistent with Cheyne–Stokes (CSB) was identified, with cycle lengths ranging from 40 to 50 s. There was a clear positional component, with central events being mode frequent in the supine position. A moderate OSA component was also present with an obstructive AHI of 10.3/h; the remaining events were classified as mixed, 10.9/h, with a total obstructive AHI of 21.2/h. Nocturnal gas exchange was markedly impaired. The oxygen desaturation index (ODI) was elevated to 58.1/h of recording, and desaturation events followed a band-like distribution throughout the night. The lowest recorded SpO_2_ was 62%, and the hypoxic burden was 194.5% min/h.

**Figure 3:**
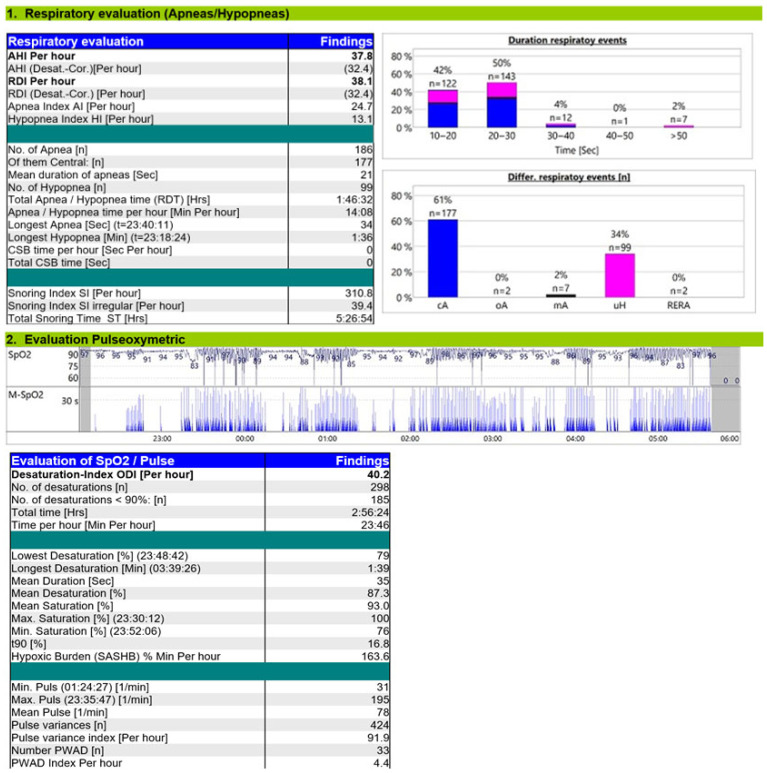
Titration study on CPAP 6 cmH_2_O.

A CPAP titration study (Figure 3) was performed in the hospital using a Löwenstein device and the SleepDocPorti 9 polygraphy system. The pressure was set at 6 cmH_2_O, based on departmental practice, with the ramp feature turned off due to nocturia. An oronasal mask was used. The patient was compliant during the study, with a total CPAP usage duration of approximately 7 h. CPAP therapy resulted in a 46% AHI reduction, with a residual AHI of 37.8/h. Central apneas remained predominant, maintaining a CSB pattern, though event duration decreased. Obstructive hypopneas emerged, often identified by snoring. Nocturnal hypoxemia improved modestly (reduced T90%), but hypoxic burden remained elevated. The device report showed no significant mask leakage.

Given the modest response to CPAP therapy, alternative treatment options were considered. The patient was deemed a suitable candidate for ASV therapy, based on a high residual central AHI, persistent CSB pattern, good compliance with PAP therapy, and preserved ejection fraction.

The patient was discharged with recommendations to continue cardiac therapy, oral iron supplementation, sleep hygiene, and positional therapy, along with home CPAP at 6 cmH_2_O using an oronasal mask. A 3-week follow-up was scheduled to assess compliance and treatment efficacy.

### 2.2. Postoperative Complications Following TURBT

The patient missed the scheduled follow-up and reported infrequent CPAP use with episodes of gasping during use. Four weeks post-discharge from the pneumology ward, TURBT was performed due to persistent hematuria and dysuria under general anesthesia. Postoperatively, he developed spontaneous breathing difficulties, resulting in delayed extubation and requiring non-invasive ventilation (NIV) for one day due to hypoxemia and reduced respiratory effort.

### 2.3. Readmission and ASV Titration Results and Settings

Six weeks later, the patient was readmitted with nocturnal gasping, insomnia, and fatigue. Although he had lost weight, abdominal obesity persisted (BMI 31 kg/m^2^). On examination, skin pallor, lethargy, and irregular tachycardia were noted.

Hematologic evaluation revealed severe iron-deficiency anemia (Hb 7.2 g/dL), and parenteral iron therapy was initiated.

A cardiac workup showed elevated NT-proBNP (1430 pg/mL) and decompensated HFpEF (EF 55%) with atrial fibrillation. The onset of atrial fibrillation is unclear, and the patient presents with a high ventricular rate (110 bpm). The patient was already on the maximum recommended dose of beta-blocker (bisoprolol 2 × 5 mg/day), and oral amiodarone was initiated for rate control. Prior to admission, the patient had received intermittent oral furosemide (40 mg every 2 days). Upon hospitalization, an intravenous loop diuretic (furosemide 40 mg IV once daily) was continued, alongside antihypertensive therapy. Therapeutic low-molecular-weight heparin (LMWH) was initiated, with hemoglobin monitoring. Blood pressure, urine output, and ventricular rate were closely monitored. Rhythm conversion was deferred until correction of the concomitant anemic syndrome.

The compliance report of the CPAP device showed a significant residual AHI of 23/h, average daily usage < 4 h, without significant leaks; evaluating the patient using an ASV device was the next treatment step. We prescribed an ASV device (Löwenstein Prisma CR), and the mode was ASVauto (automatically adjusted between min and max EPAP-dynamic); the settings were: minimum EPAP 4 cmH_2_O, maximum EPAP 10 cmH_2_O, and minimum pressure support (PS) 0 cmH_2_O, max PS of 10 cmH_2_O, back-up rate 13/min.

**Figure 4:**
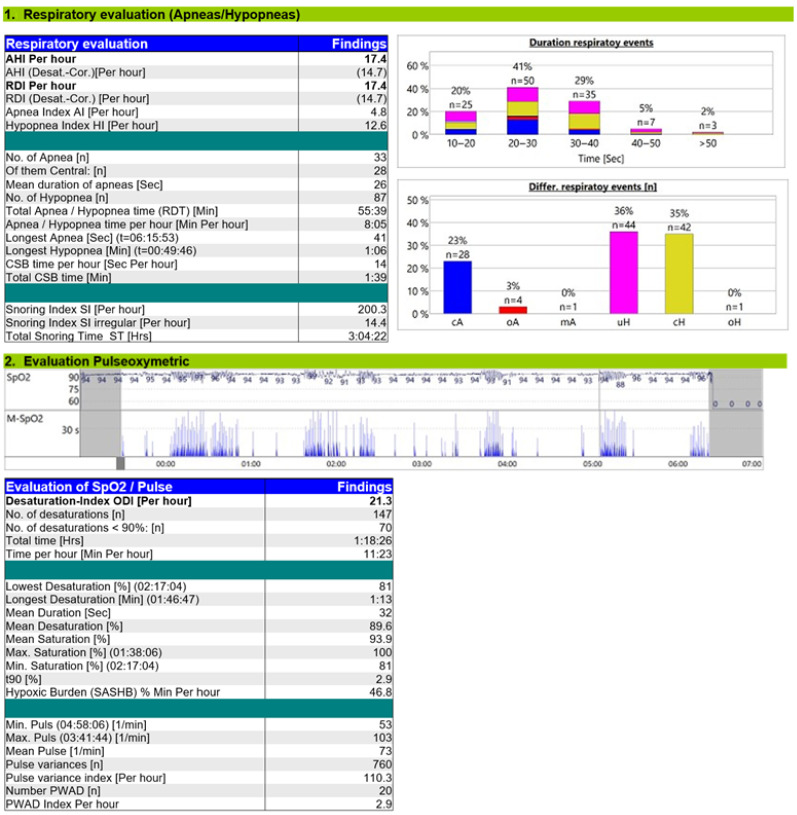
Titration study ASV.

The ASV titration study (Figure 4) demonstrated a significant reduction in CSA-CSB, with a residual AHI of 17.4/h, including a central AHI of 10.5/h. Most of the respiratory events were hypopneas. Under ASV, the oxygen desaturation index (ODI) decreased to 21/h, T90% to 2.9%, and hypoxic burden to 46.8% min/h, values approaching normal, although mild intermittent desaturations persisted.

Body position remained influential, with a supine-to-non-supine AHI ratio of 5.64:1. Device compliance data (Appendix A) showed optimal usage of 8 h/night, a residual AHI of 2/h, mandatory breaths comprising 10% of total breaths (target < 10%), a breathing stability index (BSI) of 65 (target > 60), and low leak rates (95th percentile: 17.5 L/min).

Overall, ASV therapy improved both the patient’s compliance and sleep quality.

The patient was discharged with a treatment plan indicating auto-adaptive servo-ventilation (ASV) therapy, with EPAP set to a minimum of 6 cmH_2_O, and a maximum of 12 cmH_2_O, to address residual obstructive events, along with PS ranging from 0 to 8 cmH_2_O. Additionally, positional therapy was advised, and management of heart failure with preserved ejection fraction (HFpEF), atrial fibrillation, and anemia was continued. A follow-up visit was scheduled in 4 weeks to evaluate compliance and treatment effectiveness. The patient’s progress and response to therapy will be closely monitored.

## 3. Discussion

This case represents a complex CSA-CSB phenotype, including multiple factors: heart failure with preserved ejection fraction (HFpEF), atrial fibrillation (AFib), anemia, positional influences, and mixed insomnia. These factors interact a high loop gain (LG) phenotype, characterized by ventilatory overshoot and unstable respiratory control [3].

Pathophysiologically, increased controller gain (CG)—due to enhanced chemosensitivity in AFib and anemia—led to excessive ventilation and hypocapnia. Plant gain (PG) was likely elevated by rostral fluid shifts in the supine position and reduced functional residual capacity from pulmonary congestion. While mixing gain (MG) is typically more prominent in HFrEF, our patient’s Cheyne–Stokes cycle length of 40–50 s suggests milder circulatory delay, consistent with preserved ejection fraction [3,5].

The relationship between atrial fibrillation (AFib) and central sleep apnea (CSA) is bidirectional. Increased left atrial pressure associated with AFib enhances circulatory delay and chemoreflex gain, resulting in excessive ventilatory responsiveness and promoting Cheyne–Stokes breathing (CSB). Conversely, CSB exacerbates autonomic imbalance, which may trigger or sustain AFib episodes. Effective management of AFib—such as through rate or rhythm control—has been shown to reduce the severity of CSA-CSB. Moreover, utilization of CPAP in the context of OSA lowers the risk of AFib recurrence. Bitter et al. evaluated the prevalence of CSA in patients with heart failure with preserved ejection fraction (HFpEF) and AFib and found that CSA was present in 31% of the 150 patients studied [5,6].

Anemia is another potential factor contributing to the CSA-CSB disorder; at readmission, the patient’s hemoglobin level was 7.2 g/dL. Experimental studies evaluating ventilatory responses to transient hypoxia in anemic animals showed that a hemoglobin level below 7 g/dL leads to a consistent increase in ventilation due to enhanced chemosensitivity to hypoxia. This results in increased controller gain (CG) and a narrowing of the CO_2_ reserve. The medical literature supports a correlation between obstructive sleep apnea (OSA), anemia, and heart failure. Zilberman et al. demonstrated that correcting hemoglobin levels by an average of 2 g/dL led to a reduction in the AHI by 26.5% for OSA, 29.8% for central sleep apnea, and 31.2% for Cheyne–Stokes breathing, along with improvements in minimum SpO_2_, daytime sleepiness, and New York Heart Association (NYHA) functional class [7,8,9].

In patients with CSA, positional dependency has been demonstrated in heart failure populations. A study by Szollosi et al. showed that lateral positioning significantly reduced AHI in all sleep stages, for example, from 54.7 to 27.2 events/h in Stage 1 and from 43.3 to 14.4 events/h in Stage 2. Oxygen desaturation also improved (4.7% to 3.0%), independent of apnea type or event duration, suggesting a mechanism beyond upper airway mechanics—possibly related to improved pulmonary oxygen stores. Positional therapy is a treatment option even for patients with CSA [10].

Mixed insomnia, with frequent arousals and transitions between wakefulness and light sleep, may have further destabilized respiratory control by fluctuating the CO_2_ threshold. While this may reflect a primary sleep disorder, it could also be secondary to nocturia, heart failure, or other underlying pathology. Comorbid insomnia and sleep apnea (COMISA) present as a therapy challenge because of non-compliance with CPAP therapy. To address these multifactorial contributors, integrated approaches that combine CPAP with cognitive behavioral therapy for insomnia (CBT-I) have been shown to enhance treatment adherence and improve long-term outcomes [11].

Several elements contributing to the complexity of this case have been previously discussed; however, an additional factor is the challenge of obtaining an accurate diagnosis using a surrogate monitoring system—cardiorespiratory polygraphy—instead of the gold-standard polysomnography, particularly for evaluating sleep fragmentation and correct identification of central hypopneas [2].

In our case, optimizing the medical management of the associated cardiac conditions and initiation of CPAP were the primary therapeutic steps. This included interventions for HFpEF and atrial fibrillation aimed at reducing pulmonary congestion, decreasing sympathetic drive, lowering left ventricular afterload, and controlling heart rate using diuretics, angiotensin-converting enzyme inhibitors, and antiarrhythmic medications [3,7].

Initiation of CPAP therapy was performed at a low pressure setting of 6 cmH_2_O, which represents the standard initial titration level preferred in our department for patients with CSA-CSB. This method establishes upper airway patency and reduces plant gain (PG) by decreasing ventilation/perfusion (V/Q) mismatch and increasing functional residual capacity. However, residual CSA and low compliance with CPAP therapy are common [3].

The persistence of significant CSA-CSB (residual AHI > 15/h) and a hypoxic burden of 163.6% min/h on CPAP at 6 cmH_2_O indicated the need for further treatment evaluation. The assessment of PAP therapy effectiveness is based not only on the abolition of cyclical apnea–hyperpnea patterns and the improvement of overall sleep quality, but also on the reduction of the hypoxic burden [12].

Hypoxic burden (HB) is a novel parameter designed to quantify the severity of oxygen desaturations by calculating the sum of individual areas under the oxygen saturation curve from a pre-event baseline. Unlike the oxygen desaturation index (ODI), HB considers the frequency, depth, and duration of desaturation events. Although mainly studied in OSA, HB is increasingly used to stratify cardiovascular risk. Compared to AHI, HB shows a stronger correlation with heart failure, stroke, major cardiovascular events, blood pressure, and kidney disease, and its validity has been confirmed in several clinical trials. HB is expressed as % min/h of sleep. However, the literature is scarce regarding clear thresholds for intervention or severity classification in OSA. In two large observational studies—the Osteoporotic Fractures in Men Study and the Sleep Heart Health Study—Azarbarzin et al. found that HB levels between 50 and 88% min/h are associated with an increased risk of major adverse cardiovascular events. Pinilla et al. validated this correlation in a post hoc analysis of 726 OSA patients with acute coronary syndrome, showing that patients with high HB (>75% min/h) experienced significant reductions in cardiovascular events when treated. Notably, patients with low HB (<75% min/h) showed an upward trend in cardiovascular risk compared to those receiving usual care, highlighting the dose-dependent and individualized impact of HB on CPAP indication [12,13,14].

In the present case, a threshold of <50% min/h was considered adequate for acceptable PAP therapy and cardiovascular risk reduction.

Given the persistence of ventilatory instability and CSA-CSB, in accordance with the European Sleep Society guidelines for CSA management, a trial of adaptive servo-ventilation (ASV) or an anti-cyclic pressure support device is suggested. ASV may be more effective than CPAP in treating Cheyne–Stokes breathing because it uses computer-generated algorithms to track the rhythmic fluctuations of tidal volume and dynamically adjusts pressures on a breath-by-breath basis. The device used in this case was a Löwenstein Prisma CR with ASV auto mode [15].

The ASV titration study revealed a reduction of central AHI to 10.5/h, and most residual respiratory events were central hypopneas. The tracing showed no distortion of respiratory waveforms, ataxic breathing, or bradypnea, excluding patient–ventilator desynchrony. Residual hypopneas were managed by reducing pressure support and increasing EPAP, as these events may result from an excessive anti-cyclic ventilatory response. Regarding hypoxemia, ASV therapy reduced t90%to 2.9 and hypoxic burden to 46.8% min/h. The evaluation of other parameters, such as the portion of mandatory breaths (10%) with a target of < 10%, indicated a low rate of triggered breaths and stable breathing with minimal variability, reflecting effective central respiratory control [15].

ASV has a clear indication in heart failure with preserved ejection fraction (EF > 45%) and CSA and is superior to CPAP in treating periodic breathing. Most clinical studies evaluating ASV are evaluating patients with reduce ejection fraction (LVEF < 45%); key trials include CAT-HF, ADVENT-HF, and SERVE-HF. While these studies showed that ASV improved respiratory parameters, e.g., reducing t90% from 12.5% to 2.3% in ADVENT-HF, the cardiovascular benefit is absent, most likely due to severe cardiac dysfunction. This limitation may not apply to our patient, who has preserved ejection fraction. Moreover, a meta-analysis of 2208 patients found that ASV significantly reduced the risk of major cardiovascular events in patients with LVEF > 33% or NYHA I/II. Therefore, extrapolation of ASV’s beneficial effects on oxygenation, such as t90% reduction and even hypoxic burden, remains clinically meaningful in this context. Current guidelines recommend against routine use of ASV in patients with LVEF < 45%, and if used, it should be with high caution and close monitoring [16,17,18].

Another advantage of ASV over CPAP is its potential efficacy in patients with comorbid insomnia, particularly when standard CPAP therapy has failed because of poor tolerance. This is especially relevant in our case, where complex sleep-disordered breathing coexists with persistent insomnia and likely contributes to therapy non-adherence. In a randomized controlled trial of 40 patients with chronic treatment-resistant insomnia, who were later identified as having undiagnosed OSA, ASV demonstrated a significant reduction in insomnia severity compared to CPAP (Hedges’ g = 2.5 vs. 1.39) and better remission rates of insomnia (68% vs. 24%). These findings suggest that the superior efficacy of ASV may be mediated by its ability to reduce expiratory pressure intolerance, stabilize ventilation, and minimize sleep fragmentation. Given the similar clinical phenotype in our patient, ASV may not only improve oxygenation and respiratory events, but also restore sleep continuity and reduce arousal-driven insomnia, thereby enhancing overall treatment adherence [19].

Additional interventions to reduce anti-cyclic ventilator activity and improve outcomes include supplemental oxygen, non-supine positioning, or use of a non-vented mask to minimize hypocapnia, administration of carbonic anhydrase inhibitors (e.g., acetazolamide), or promoting stable non-REM sleep using sedatives or hypnotics (e.g., zolpidem). Nocturnal oxygen therapy may be a practical option in cases of CPAP failure, especially for non-hypercapnic CSA, by reducing controller gain. A meta-analysis by Ruan et al. showed that oxygen therapy alone reduced AHI by 31% and increased SpO_2_ by 3% compared to baseline [2,20].

Considering these results, reassessment of CSA-CSB management after addressing the aforementioned factors and patient adaptation to ASV therapy is recommended. This should include repeat titration studies and evaluation with polysomnography.

From the patient’s perspective, it is too early to assess the full clinical efficacy of adaptive servo-ventilation (ASV) therapy. The patient understands that over time, consistent use of ASV may help preserve cardiac function and reduce cognitive decline associated with intermittent hypoxia, ultimately contributing to a longer and more active life.

## 4. Conclusions

Central sleep apnea with Cheyne–Stokes breathing (CSA-CSB) presents a significant therapeutic challenge in patients with heart failure with preserved ejection fraction (HFpEF) and concurrent anemia. Effective management of these comorbidities can mitigate the severity of CSA-CSB. Given the intricate pathophysiology of CSA-CSB, characterized by ventilatory instability and high loop gain, precise diagnosis and individualized treatment strategies are essential to optimize patient outcomes.

In this case, ASV proved effective after CPAP therapy failure, resulting in improved adherence, a significant reduction in hypoxic burden, and enhanced sleep quality. While these short-term benefits are encouraging, the long-term impact on atrial fibrillation recurrence, cardiac function improvement, and insomnia severity requires ongoing evaluation.

## Figures and Tables

**Figure A1:**
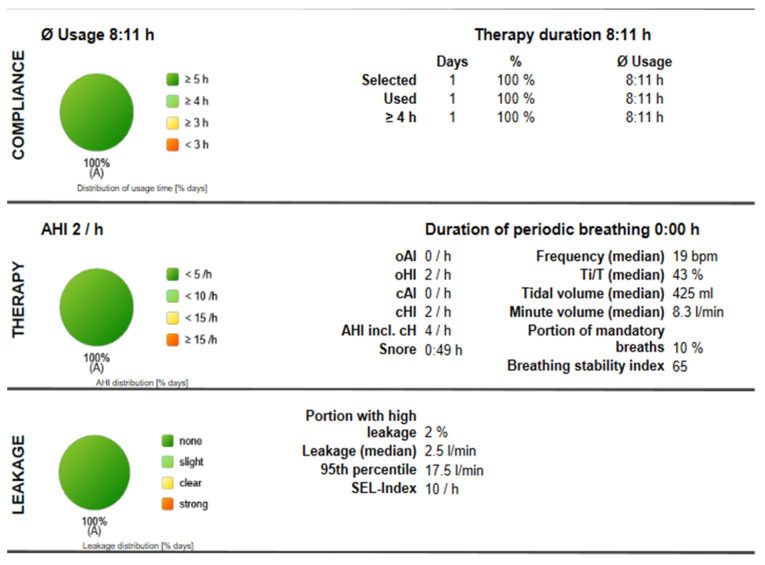
Compliance Report for Titration Using ASV.

## Data Availability

The original contributions presented in this study are included in the article. Further inquiries can be directed to the corresponding author.
